# Sleep Compensation on Work-Free days Is Associated with Better Sleep Quality

**DOI:** 10.2147/NSS.S562192

**Published:** 2025-12-16

**Authors:** Diana Aslamyar, Charlotte von Gall

**Affiliations:** 1 Institute of Anatomy II, Medical Faculty, Heinrich Heine University, Düsseldorf, Germany

**Keywords:** REM sleep, chronotype, social jet lag, sleep efficiency, PHQ-4, workload, PSQI

## Abstract

**Purpose:**

Sleep of sufficient duration and quality is crucial for physical and mental health and performance. In modern urban society, work-related factors such as perceived workload and limited sleep duration on workdays appear to have a significant impact on sleep and well-being. Our current study examines both subjective and objective measures of sleep on workdays and work-free, as well as their associations.

**Methods:**

The study combines questionnaires and longitudinal wearable (Fitbit Inspire 2) sleep data from young, healthy adults (aged 18–48 years) in a German metropolitan region (n = 67). Associations were investigated by Spearman correlation analyses with a 95% confidence interval.

**Results:**

Perceived workload was associated with symptoms of depression/anxiety, daytime dysfunction, as well as subjective sleep quality, in particular on workdays. Sleep and sleep stages were objectively longer and later on work-free days than on work-days. Likewise, objective sleep efficiency and subjective sleep quality were higher on work-free days. Longer sleep duration, thus sleep compensation, on work-free days was associated with subjectively more restful and better sleep, and objectively with later phase and a higher proportion of REM sleep.

**Conclusion:**

These data suggest that work-related sleep loss should be avoided whenever possible or at least compensated for on work-free days in order to achieve not only sufficient sleep duration but also good sleep quality, which is pivotal for mental health and performance.

## Introduction

Restful sleep is essential for regeneration and energy balance and plays an important role for mental- and physical health.^1–8^ Insufficient sleep is manifested by daytime fatigue and reduced performance and can lead to increased stress and symptoms of anxiety and depression.^1–8^ Sleep quantity and quality are interrelated components of restful sleep.^9^ Both contribute equally to physical and mental well-being^6^,^10–12^ and are negatively associated with workload.^11^ In particular, short sleep duration on workdays appears to affect the subjective perception of workload, which in turn is associated with symptoms of depression.^13^

The required sleep duration for adults is somewhere between 7–9 hours,^14^ but shows large individual differences. In addition, there are both extrinsic and intrinsic factors^15^ that influence bedtime and thus also sleep duration and quality. Intrinsic components include the homeostatic sleep drive as well as a circadian component.^16^,^17^ The individual phase of the sleep propensity rhythm is essentially controlled by an internal clock^18^ and can also be used to determine the chronotype.^19–22^ Particularly in modern urban society,^23^ extrinsic factors include artificial light in the evening, which can delay bedtime, and the early start of work, which can be associated with premature end to sleep. Sleep deficits due to getting up early on workdays often cannot be counterbalanced simply by going to bed earlier. Due to the intrinsic phase of the sleep propensity rhythm, an earlier bedtime may just lead to a longer sleep latency if the homeostatic sleep pressure is not high enough. However, work-free days can be used to compensate for the accumulated sleep dept. Sleep compensation is probably one of the reasons why sleep on work-free days is perceived as much better than on workdays.^24–27^

Measuring objective sleep quality still presents a major challenge. Because sleep continuity is a major hallmark of sleep quality, the time spent awake after sleep onset (WASO) and the ratio of total sleep time to time spent in bed, which is referred to as sleep efficiency, provide qualitative measures. In addition, sleep architecture is also of particular importance for sleep quality. Each sleep cycle consists of a sequence of distinct sleep stages characterized by fluctuations in muscle tone, brain activity patterns, heart rate, body temperature, and eye movements.^28^ Each sleep cycle starts with brief transitions between wakefulness and light sleep (stage 1), followed by light sleep (stage 2) and deep sleep (stage 3) and ends with rapid eye movement (REM) sleep. Each cycle lasts roughly 90–120 minutes and repeats 4–6 times per night. Over the course of the night, the length of the stages changes, with deep sleep decreasing and REM sleep increasing. Deep sleep and REM sleep each account for about a quarter of total sleep.^28^ REM sleep appears to be particularly important for sleep quality.^27^,^29^,^30^ Since REM sleep occurs predominantly in the second half of the night and the early morning,^31^,^32^ it seems to be particularly affected by being woken up by the alarm clock in the early morning.^27^,^33^ Sleep stages can be precisely determined using polysomnography, which typically requires a visit to a sleep laboratory. However, they can also be assessed by algorithms based on heart rate and movement patterns, using wrist-worn wearables. Analysis of sleep and sleep stages such as deep sleep, WASO and REM sleep by Fitbit wearables is less accurate than polysomnography,^34–39^ but can be effectively used in field studies.^27^,^33^ Fitbit Alta HR and Inspire HR, the predecessors of Fitbit Inspire 2 used in our study, shows comparatively good specificity and sensitivity for sleep and sleep stages.^35^,^39^ A meta-analysis shows that Fitbit wearables in particular exhibit a high degree of comparability with polysomnography in terms of key sleep parameters such as sleep duration, WASO, and sleep efficiency.^40^ Fitbit-derived sleep data have been used in epidemiological research, showing strong associations between sleep quality and health outcomes.^41^

Previous studies have examined differences in sleep quality between workdays and work-free days based on either standardized questionnaires^24^,^25^ or wearable devices,^27^ but not yet combined. Our hypothesis in this study was that there is an association between subjective sleep quality on working days and work-free days determined by questionnaire and the corresponding objectively measured longitudinal sleep data from a Fitbit wearable device. Our particular focus is on better understanding the differences of key sleep parameters such as sleep duration, WASO, sleep efficiency and REM sleep between workdays and work-free days as a basis for improving sleep and thus performance and well-being.

## Materials and Methods

### Procedure and Participants

Part of the data used for this study came from the same dataset as previously described.^24^ The participants were recruited in the Düsseldorf metropolitan region between 5 May 2023 and 18 September 2024 through personal contacts using a snowball system. Criteria for recruitment included age between 18 and 50, regular weekly working hours (including school/university), no shift work and no self-reported chronic illness (including sleep disorders).

All participants were informed about the study conditions and data protection regulations and gave their consent before study enrolment. Participants were equipped with Fitbit Inspire 2 multisensory (movement and heart rate) sleep trackers and asked to wear them as continuously as possible for three months, especially at night. The data collection period includes 64 days of the week, defined as working days, and 26 weekend days, defined as non-working days. After the data collection period, participants were asked to complete the online questionnaire where all questions were mandatory and could only be answered once. They were then automatically redirected to the Fitbit website, where they could authorize the transfer of activity and sleep data to our study server.

The study was conducted in accordance with the ethical requirements of the Declaration of Helsinki and approved by the Research Ethics Committee of the Faculty of Medicine of Heinrich Heine University (file number of approval: 2019–3786).

### Questionnaire

The online questionnaire contained questions addressing general characteristics such as biological sex, age, body weight, height, the inclusion criteria and lifestyle factors.

The questions on work asked whether the participants had regular employment, whether they worked on weekends, whether they worked shifts, and how high they generally rate their workload on a four-point scale (low=0, moderate=1, high=2, very high=3).^13^,^27^

The questions about health included the 4-item patient health questionnaire for depression and anxiety (PHQ-4), according to Lowe et al (2010),^42^ each scored on a four-point scale (not at all=0, on single days=1, on more than half of the days=2, almost every day=3).

Self-reported sleep quality was assessed with a question on how tired participants felt when waking in the morning on a three-point scale (rested=0, tired=1, very tired=2)^24^ and with the Pittsburgh Sleep Quality Index (PSQI).^30^ These were asked separately for workdays and work-free days.^24^,^25^ The seven PSQI components, each assessed on a four-point scale^24^ and reflecting a different dimension of sleep, were summed to produce the global scores (range 0–21) and also considered separately. Lower scores indicate better sleep.

The consumption of alcohol and caffeinated drinks, as well as physical activity, were rated on a five-point scale (none = 0, 1–2 times per week = 1, several times per week = 2, once daily = 3, several times daily = 4), while drug use and consumption of nicotine (=smoking cigarettes/e-cigarettes) were rated on a two-point scale (no = 0, yes = 1).

### Assessment of Activity, Sleep and Sleep Stages Based on Fitbit Wearable Data

Activity and sleep were assessed by Fitbit analyses over a period of three month. Proprietary Fitbit algorithms detect activity,^43^ sleep and sleep stages^44^ based on heartrate and activity patterns. A python-based custom software application was used to process Fitbit data. The variables were determined for the entire period (total) as well as separately for workdays and work-free days. The phases of total sleep and sleep stages were assessed based on their midpoint in local time (digital).

### Data Analysis

Statistical analysis was performed using Prism Version 7.01 (GraphPad) and R studio Version 2023.12.0+369. Normal distribution of data was tested by D’Agostino and Pearson normality test. For descriptive statistics, the mean ± standard error of the mean was used for normally distributed data while the median with the interquartile range was used for non-normally distributed data.

Because not all variables were normally distributed, non-parametric tests were used consistently. Statistical significance was assumed at P<0.05. The Wilcoxon matched-pairs signed rank test was used to compare variables between workdays and work-free days. The strength and direction of relationships between categorial and continuous variables were analyzed by Spearman’s rank correlation analysis with a 95% confidence interval (CI). The strength and direction of relationships between a “yes/no” variable and continuous variables were analyzed by Point-biserial correlation with a 95% CI. The Holm-Bonferroni correction was used to reduce the probability of Type I errors in multiple correlations. Linear regression was used for graphical visualization of relationships, even if data were not normally distributed.

## Results

### Sample Size

Of the 75 subjects we recruited, we had to exclude 3 subjects because they met at least one exclusion criterion. 5 subjects were not included because they did not reach the cut-off of wearing the Fitbit tracker in 50% of the nights throughout the study period. This resulted in a total sample size of n=67. Based on this sample size and an estimated effect size of 0.4, a statistical power of 0.97 was achieved (G*Power).

### General Characteristics, Depression/Anxiety and Workload Perception

The distribution of age, sex and BMI is summarized in Table 1. The median age was 25 (21–25) years, 39 were female and 28 male. The median BMI was 22.6 (21.2–25.6) kg/m^2^. The majority (61%) of participants were in the normal weight range (18.5–24.9 kg/m^2^) according to the WHO classifications.^45^

**Table 1:** Sex, Age, and BMI (n=67)

	n	% of Total
Sex		
Female	39	58
Male	28	42
Age (years)		
18-27	42	63
28-37	16	24
38-47	9	13
BMI (kg/m^2^)		
<18.5	5	7
18.5–24.9	41	61
25.0–29.9	12	18
30.0–34.9	8	12
35.0–39.9	1	1
≥ 40.0	0	0

The distribution of the PHQ-4 score and the score for perceived workload is shown in Table 2. The majority of participants reported having a moderate (27%) to high (40%) workload. The majority (46%) of participants had a PHQ-4 score (depression/anxiety) in the normal range (0–2), followed by 39% in the mild range (3–5), according to Lowe et al (2010).^42^ Depression/anxiety and perception of workload were not significantly associated with general characteristics of the sample but with each other (Supplementary Table 1).

**Table 2:** PHQ-4 (Depression/Anxiety) and Workload Perception (n=67)

	n	% of Total
PHQ-4 score		
0-2	31	46
3-5	26	39
6-8	7	10
9-12	3	4
Workload score		
Low	15	22
Moderate	18	27
High	27	40
Very high	7	10

### Differences in Self-Reported and Wearable Measured Sleep Variables Between Workdays and Work-Free days

Table 3 summarizes the differences in self-reported sleep variables between workdays and work-free days. The global PSQI score was significantly higher on workdays than on work-free days, indicating poorer sleep on workdays. Consistently, the scores for tiredness on waking, C1 (subjective sleep quality), C2 (sleep latency), C3 (sleep duration), and C7 (daytime dysfunction) were significantly higher on workdays than on work-free days, indicating poorer sleep quality and quantity, and worse daytime performance on workdays. Tiredness on waking, subjective sleep quality and daytime dysfunction were associated with each other (Supplementary Table 2).

**Table 3:** Differences in Self-Reported Sleep Variables Between Workdays and Work-Free days in the Sample (n=67)

	Median	IR	P value
Tiredness on waking			0.0005***
Workdays	2	[1,2]	
Work-free days	1	[1,2]	
PSQI global			<0.0001****
Workdays	6	[4,7]	
Work-free days	4	[1,2]	
PSQI components			
C1 Subjective sleep quality			<0.0001****
Workdays	1	[1,2]	
Work-free days	1	[0,1]	
C2 Sleep latency			0.0035**
Workdays	1	[1,1]	
Work-free days	1	[0,1]	
C3 Sleep duration			<0.0001****
Workdays	1	[1,2]	
Work-free days	0	[0,1]	
C4 Sleep efficiency			0.16
Workdays	0	[0,0]	
Work-free days	0	[0,0]	
C5 Sleep disturbances			0.71
Workdays	1	[1,1]	
Work-free days	1	[1,1]	
C6 Use of sleep medication			0.08
Workdays	0	[0,0]	
Work-free days	0	[0,0]	
C7 Daytime dysfunction			0.004**
Workdays	1	[0,2]	
Work-free days	1	[0,1]	

**Notes**: Wilcoxon matched pairs signed rank test. **P<0.01, ***P<0.001, ****P<0.0001, higher values on work-free days.

**Abbreviations**: IR, interquartile range; PSQI, Pittsburgh Sleep Quality Index.

The differences in Fitbit-measured sleep variables between workdays and work-free days are shown in Table 4. The phases of total sleep, deep sleep REM sleep, and WASO were significantly later on work-free days than on workdays. In addition, the duration of total sleep, deep sleep REM sleep, and WASO were significantly longer on work-free days than on workdays. The proportion of REM sleep was significantly higher on work-free days than on workdays while the proportion of WASO was significantly lower on work-free days than on workdays. Sleep latency was lower and sleep efficiency was higher on work-free days. There was no significant difference in activity based on calculated calorie-consumption between workdays and work-free days (P=0.24, n=67).

**Table 4:** Differences in Fitbit-Measured Sleep Variables Between Workdays and Work-Free days (n=67)

	Median	IR	P value
Phase (digital local time)			
Total sleep			0.0001****
Workdays	4.2	[3.1,4.9]	
Work-free days	5.3	[4.6,6.2]	
Deep sleep			0.0001****
Workdays	2.6	[1.6,3.9]	
Work-free days	3.4	[2.5,4.6]	
REM sleep			0.0001****
Workdays	5.3	[4.4,6]	
Work-free days	6.2	[5.2,7]	
WASO			0.0001****
Workdays	4.3	[3.1,5.4]	
Work-free days	5.7	[4.3,7]	
Duration (min)			
Total sleep			0.0001****
Workdays	446	[412,487]	
Work-free days	487	[434,509]	
Deep sleep			0.0001****
Workdays	70	[63,80]	
Work-free days	77	[65,87]	
REM sleep			0.0001****
Workdays	83	[66,95]	
Work-free days	87	[74,148]	
WASO			0.01*
Workdays	56	[51, 62]	
Work-free days	58	52,66]	
Proportion (%)			
Deep sleep			0.48
Workdays	16	[14,18]	
Work-free days	16	[14,18]	
REM sleep			0.03*
Workdays	18	[16,20]	
Work-free days	18	[16,20]	
WASO			0.008^++^
Workdays	12	[12,14]	
Work-free days	12	[12,13]	
Sleep latency (min)			0.0001^++++^
Workdays	6	[4,7]	
Work-free days	5	[3, 6]	
Sleep efficiency (%)			0.04*
Workdays	88	[86, 88]	
Work-free days	89	[87, 89]	

**Notes**: Wilcoxon matched pairs signed rank test. *P<0.05; ****P<0.0001, higher (later) values on work-free days. ^++^P<0.01; ^++++^P>0.0001, lower values on work-free days.

**Abbreviations**: IR, interquartile range; min, minutes; REM, rapid eye movement; WASO, wake after sleep onset.

### Associations Among Subjective Sleep Quality, Daytime Dysfunction, Depression/Anxiety, and Workload Perception

**Figure 1:**
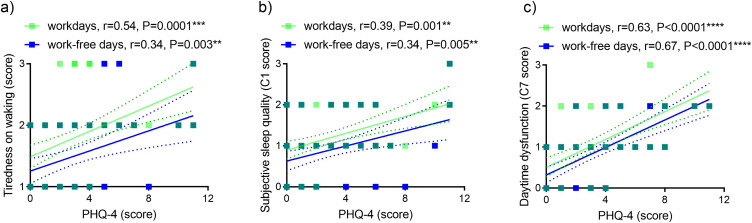
Relationship between depression/anxiety and self-reported sleep variables. Depression/anxiety (PHQ-4) and (**a**) tiredness on waking, (**b**) subjective sleep quality (C1), (**c**) daytime dysfunction (C7).

**Note**: n=67, **P<0.01, ***P<0.001, ****P<0.0001, green symbols semi-transparent. Dashed lines, 95% confidence intervals.

**Abbreviations**: C1, component 1 of PSQI; C7, component 7 of PSQI; PHQ-4, Patient Health Questionnaire-4; PSQI Pittsburgh Sleep Quality Index; r, Spearman correlation coefficient.

Tiredness on waking, poor subjective sleep quality (C1) and daytime dysfunction (C7) correlated positively with each other (Table 2). PHQ-4 (depression/anxiety) correlated positively with Tiredness on waking, poor subjective sleep quality (C1) and daytime dysfunction (C7) on workdays and work-free days (Figure 1).

**Figure 2:**
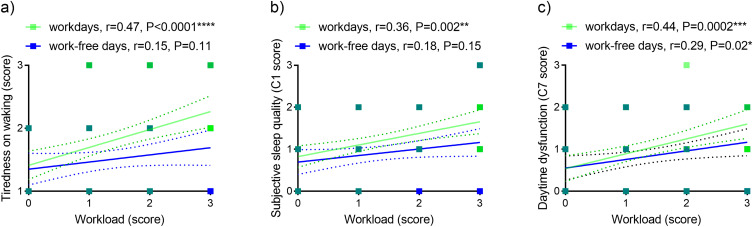
Relationship between workload perception and self-reported sleep variables. Workload and (**a**) tiredness on waking, (**b**) subjective sleep quality (C1), (**c**) daytime dysfunction (C2).

**Note**: n=67, *P<0.05; **P<0.01, ***P<0.001, ****P<0.0001, green symbols semi-transparent. Dashed lines, 95% confidence intervals.

**Abbreviations**: C1, component 1 of PSQI; C7, component 7 of PSQI; PSQI; Pittsburgh Sleep Quality Index; r, Spearman correlation coefficient.

Perception of workload correlated positively with tiredness on waking and poor subjective sleep quality (C1) on workdays (Figure 2a and b), and with daytime dysfunction (C7) on workdays and work-free days (Figure 2c).

### Associations Between Wearable-Measured and Self-Reported Sleep Variables

**Figure 3:**
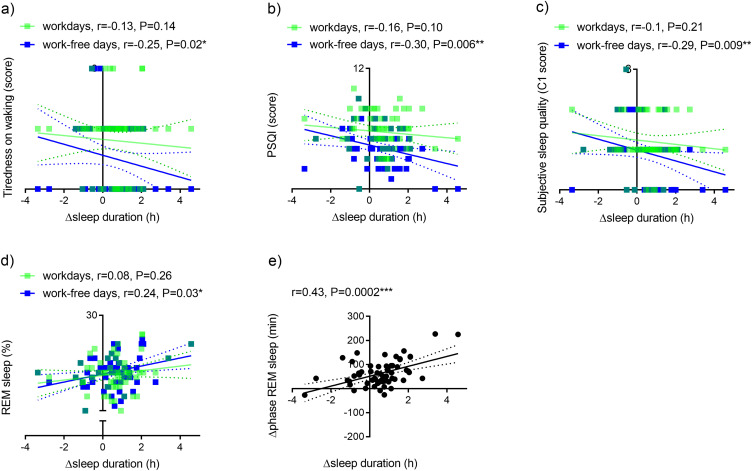
Relationship between the difference in wearable-measured sleep duration between workdays and work-free days (Δsleep duration) with self-reported sleep variables and REM sleep. Relationship between Δsleep duration and (**a**) tiredness on waking, (**b**) global PSQI, (**c**) Subjective sleep quality (C), (**d**) proportion of REM sleep, (**e**) the difference in REM sleep phase between workdays and work-free days (Δphase REM sleep).

**Note**: n=67, *P<0.05; **P<0.01, ***P<0.001, green symbols semi-transparent. Dashed lines, 95% confidence intervals.

**Abbreviations**: C1, component 1 of PSQI; PSQI, Pittsburgh Sleep Quality Index; r, Spearman correlation coefficient; REM, rapid eye movement.

First, we investigated the relationship between objective measures for sleep duration with self-reported sleep quality. The difference in wearable-measured sleep duration between workdays and work-free days (Δsleep duration) correlated negatively with tiredness on waking (Figure 3a), global PSQI scores (Figure 3b) and poor subjective sleep quality (C1) (Figure 3c).

Next, we investigated the relationship between (Δsleep duration) and wearable-measured REM sleep proportion and the difference in REM sleep phase between workdays and work-free days (Δphase REM sleep). Δsleep duration correlated positively with REM sleep proportion (Figure 3d) on *work-free days*. Moreover, Δsleep duration correlated positively with the difference in the phase of REM sleep between workdays and work-free days (Δphase REM sleep) (Figure 3e). There was no significant correlation of Δsleep duration with the proportion (r=−0.027, P=0.83) or the Δphase (r=0.08, P=0.25) of deep sleep.

**Figure 4:**
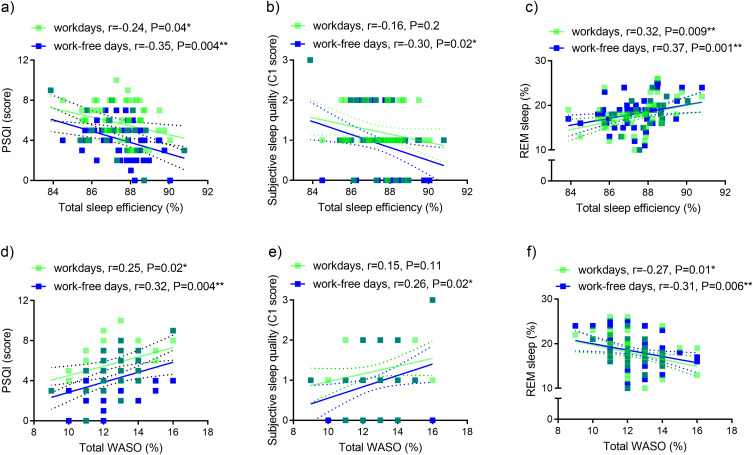
Relationship between wearable-measured and self-reported sleep variables. Associations between wearable-measured sleep efficiency of the total data collection period and (**a**) global PSQI, (**b**) C1 of PSQI, (**c**) proportion of REM sleep. Associations between WASO proportion of the total data collection period and (**d**) global PSQI, (**e**) C1 of PSQI, (**f**) proportion of REM sleep.

**Note**: n=67, *P<0.05; **P<0.01, green symbols semi-transparent. Dashed lines, 95% confidence intervals.

**Abbreviations**: C1, component 1 of PSQI, PSQI, Pittsburgh Sleep Quality Index; r, Spearman correlation coefficient; REM, rapid eye movement, WASO, wake after sleep onset.

Wearable-measured sleep efficiency of the entire data collection period (total sleep efficiency) correlated negatively with global PSQI scores on workdays and work-free days (Figure 4a) and poor subjective sleep quality (C1) on work-free days (Figure 4b) and positively with REM sleep proportion on workdays and work-free days (Figure 4c). The wearable-measured proportion of wake after sleep onset of the entire data collection period (total WASO) showed corresponding inverse relationships (Figure 4d–f).

There were no significant correlations between lifestyle variables and objectively measured sleep variables (Supplementary Table 3).

## Discussion

This study, which combines self-reported and Fitbit wearable-based data, contributes to the understanding of the differences between sleep quality on workdays and work-free, both of which contribute to mental well-being and performance.

In line with earlier studies with a general population^25^ and a larger cohort with a similar population,^24^ we also found significantly lower global PSQI scores and thus generally better sleep quality on work-free days than on workdays. The analysis of the PSQI components indicates in particular higher subjective sleep quality (C1), lower sleep latency (C2), higher sleep duration (C2), as well as a lower daytime dysfunction (C7) on work-free days, similar to previous studies.^24^,^25^ In addition, tiredness on waking, a variable we have used in previous studies as an indicator for sleep quality,^13^,^24^,^27^ was lower on workfree days. Tiredness on waking and daytime dysfunction were associated with poor subjective sleep quality, consistent with good sleep quality being important for feeling rested and performance. Similar to our previous study,^24^ perceived workload, tiredness on waking, poor subjective sleep quality, and daytime dysfunction were associated with symptoms of depression and anxiety. This highlights the role of work-related psychological stress and subjective sleep quality for mental health and performance, which in turn are directly related to general well-being.^46^ A meta-analysis demonstrates the negative impact of occupational stress on self-reported sleep quality measured by the standard PSQI^47^ which primarily reflects sleep quality on workdays.^25^ By differentiating between workdays and non-workdays, we can conclude, in line with our previous observations,^24^,^27^ that perceived workload does indeed have a stronger impact on subjective sleep quality on *workdays*. However, it appears to have a sustained impact on daytime dysfunction on all days. Thus, reducing work-related stress, for example through meditation,^47^ could help improve sleep on workdays and the feeling of functionality.

The inclusion of objective measures to assess sleep is pivotal in understanding the foundations of restful sleep. Consistent with our earlier study in young adults,^27^ the Fitbit-measured duration of sleep and sleep stages (including WASO) were longer on work-free days than on workdays. This indicates a sleep loss on workdays and a sleep compensation on work-free days. Moreover, the phase of sleep and sleep stages (including WASO) were later on work-free days, consistent with social jet lag. In addition, the Fitbit-measured sleep latency was shorter on work-free days, suggesting a higher sleep pressure. Fitbit-measured WASO was also lower on work-free days and consequently sleep efficiency was higher, indicating a better sleep consolidation. Interestingly, the proportion of REM sleep, which was inversely associated with sleep efficiency and the proportion of WASO, was higher on work-free days. This suggests that better sleep consolidation on work-free days is in favor of REM sleep, which appears to be important for sleep quality.^27^,^29^,^30^

A longer sleep duration on work-free days than on workdays was associated with less tiredness on waking, lower global PSQI score, thus self-reported sleep quality,^30^ and in particular with subjective sleep quality (C1) on *work-free days*. This suggests an association between objective sleep quantity and subjectively more restful sleep and better sleep quality. This is consistent with sleep quantity and quality being interrelated components of restful sleep.^9^ These associations also indicate that compensating for work-related sleep loss contributes to subjectively more restful and better sleep quality on work-free days. Sleep compensation appears to be particularly^48^ but not exclusively (this study) important for late chronotypes. Although official sources appreciate the negative effects of sleep loss on health and sleeping in on weekends as a common way to catch up on sleep and/or return to a normal sleep rhythm, it is often recommended to set the alarm even on weekends for consistency (eg).^49^ However, since our studies suggest that sleeping in on work-free days contributes to better sleep and thus well-being, the start of work should rather be adjusted to individual needs whenever possible.

Moreover, a longer sleep duration on work-free days than on workdays was associated with a higher proportion of REM sleep. This is consistent with REM sleep, which occurs predominantly in the second half of the night and early in the morning,^31^,^32^ is shortened by being woken up early on workdays and prolonged by sleeping in on non-workdays. Similarly, a longer sleep duration on work-free days than on workdays was associated with a later phase of REM sleep. The phase of the REM sleep appears to be strongly controlled by the circadian component of sleep timing^17^ and is therefore also discussed as a good indicator for the intrinsic phase position.^50^

We found associations between subjective sleep quality and objective measures for sleep quality in particular on *work-free* days. Higher Fitbit-measured sleep efficiency was associated with less tiredness on waking, lower global PSQI and C1 scores. Similarly, a lower WASO proportion, rather than WASO duration, was associated with less tiredness on waking, lower global PSQI and C1 scores. This is consistent with the associated between perception of insomnia and restless sleep with critical transitions in the sleep architecture.^51^ Thus, sleep efficiency and WASO proportion, which can be measured easily using a variety of commercial wearable sleep-tracking devices^39^,^48^ appear to be good digital markers for sleep quality, in particular in the absence of workday constraints.

Finally, we would like to emphasize that this study was conducted on healthy, younger adults from a metropolitan area who do not perform shift work. Different results are likely to be obtained in a rural population, older adults, people with chronic illnesses, or shift workers.

## Limitations

Our cohort is relatively small and does not represent a cross-section of the population. The Fitbit’s sleep stage analysis is not as accurate as polysomnography. Furthermore, correlations provide a measure of strength and direction of relationships but not causality.

## Conclusion

Our data indicate that the feeling of sleeping well and waking up rested contributes to psychological well-being and performance. The poorer sleep quality on workdays compared to work-free days appears to be associated, in addition to work-related psychological factors, primarily to shorter sleep duration, lower sleep efficiency, and a lower proportion of REM sleep in favor of a higher proportion of WASO. Sleep compensation on work-free days appears to have a positive effect on subjective sleep quality and the proportion of REM sleep. To achieve better sleep quality on workdays, measures to increase stress resistance could be implemented and, if possible, work should be started later. Alternatively, the sleep dept should be compensated for on work-free days.

## Data Availability

Data will be made available on reasonable request from the corresponding author.
